# Comparison of Vegetarian Diets and Omnivorous Diets on Plasma Level of HDL-c: A Meta-Analysis

**DOI:** 10.1371/journal.pone.0092609

**Published:** 2014-03-26

**Authors:** Zili Zhang, Jian Wang, Sifan Chen, Zhaoyu Wei, Zhengtu Li, Siwen Zhao, Wenju Lu

**Affiliations:** 1 State Key Laboratory of Respiratory Diseases, Guangzhou Institute of Respiratory Disease, The First Affiliated Hospital, Guangzhou Medical University, Guangzhou, Guangdong, China; 2 Department of Nutrition, School of Public Health, Sun Yat-Sen University, Guangzhou, Guangdong, China; 3 Department of student affairs, Guangzhou Medical University, Guangzhou, Guangdong, China; 4 The second Affiliated Hospital, Guangzhou Medical University, Guangzhou, Guangdong, China; University of Milan, Italy

## Abstract

Low plasma level of high density lipoprotein cholesterol (HDL-c) was an independent risk factor for cardio vascular disorder, and associated with poor outcomes in pulmonary arterial hypertension. To compare the effects of vegetarian diets and omnivorous diets on HDL-c in plasma, we identified cross-sectional and cohort studies related to HDL-c listed on PubMed and ISI Web of Knowledge as well as the corresponding references (until Nov, 2013). Twelve studies with a total of 4177 individuals were selected for meta-analysis. This meta-analysis indicates that vegetarian diets did not alter plasma HDL-c concentrations, as it wasn’t initially expected by the authors [Standardized Mean Difference (SMD) = 0.02 mmol/l; 95% confidence interval (CI): −0.19 to 0.22 mmol/l]. In Asia and Latin America countries, no significant differences in HDL-c levels between vegetarians and omnivores were found (SMD = −0.09 mmol/l; 95% CI: −0.43 to 0.25 mmol/l). In Europe and North America countries, the plasma level of HDL-c was also not different between the two diets (SMD = 0.09 mmol/l; 95% CI: −0.19 to 0.36 mmol/l). In light of this meta-analysis, we conclude that there is no evidence that plasma HDL-c levels differs in vegetarians and omnivores, even after adjusting for cultural circumstances.

## Introduction

Dyslipidemia is a condition often associated with coronary heart diseases, peripheral vascular diseases, atherosclerosis, and stroke [1]–[3]. It is a common public health problem worldwide, and imposes a significant demand on medical care and health services because of its high prevalence in the general population. In United States of America, dyslipidemia and its related diseases account for over 885,000 deaths and $634.2 billion in related costs annually [4]. Compared to omnivorous subjects, vegetarians have diminished risk of developing dyslipidemia. This may be due to their lower saturated fats consumption and higher fiber intake, which results in a reduction in the concentration of low density lipoprotein cholesterol (LDL-c), a major cardiovascular disease risk factor [5]. Greater fruits, nuts, and cereals consumption may also contribute to atherosclerosis prevention by increasing the intake of anti-oxidants, vitamins, and micronutrients [6]. Moreover, a recently study when vegans were compared to omnivorous subjects also shown functional aspects of high density lipoprotein (HDL) and defective chylomicron removal and VLDL (very low-density lipoprotein) remnants from the plasma may be affected by dietary habits [7]. It is said that high triglyceride (TG), low LDL-c, and low HDL-c levels are predictive for the increased risk of dyslipidemia and are considered to be the major risk factors, followed by smoking and high blood pressure [8]–[10]. Especially, it was estimated that 11% of the US men had low HDL-c levels [11]. At present, the clinical management of dyslipidemia risk involves targeting multiple risk factors to achieve the maximum decrease in risk and HDL-c has been an important target for intervention efforts. HDL is responsible for reverse transportation of cholesterol, carrying basically the cholesterol from tissues to the liver. It has been known for quite a long time that the plasma concentration of HDL-c correlated inversely with the incidence of cardio vascular disorder (CVD) and coronary heart disease (CHD) and that low HDL-c was an independent CVD risk factor (particularly when HDL-c <1.0344 mmol/l) [8], [12], [13]. According to the Vahit Study, a 1 mg/dl HDL reduction results in 3%−4% increase in coronary artery disease (CAD) prevalence. It is now a common practice to measure HDL-c level [13] and compute the ratio of LDL-c to HDL-c or the cholesterol to HDL-c for a better assessment of CHD risk [14]. Importantly, a recent study demonstrated that low plasma level of HDL-c was associated with higher mortality and clinical worsening in pulmonary arterial hypertension patients [15].

Nowadays, with the development of society and economy, specialists began to pay much attention to the relationship of different diets and plasma cholesterol levels. In this study, vegetarian diets and omnivorous diets have been considered, and we wanted to compare whether there is a difference in HDL-c concentrations between the two kinds of diets. In view of conflicting results and the limited sample sizes in individual studies, we performed a meta-analysis for this comparison. In addition, stratified analysis was made by some cultural similarities in order to find whether different cultural background represents a factor influencing the plasma level of HDL-c.

## Methods

### Literature Search

**Figure 1:**
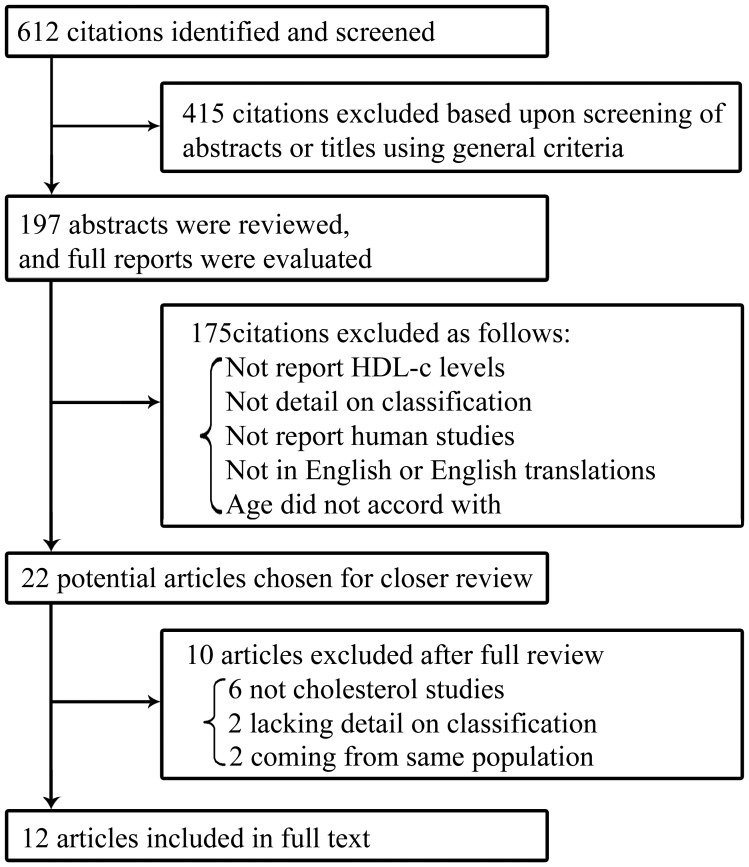
Results of search for eligible studies.

A systematic search of the literature was carried out by using Pub Med and ISI Web of Knowledge resources (until Nov, 2013). The keywords used for the search included “lacto” or “vegetarian” or “non meat eaters” or “omnivorous diet” or “omnivores” or “meat- eating” or “no vegetarian” concatenated with “blood lipid levels” or “plasma lipids” or “HDL-c” or “plasma lipoproteins”. Two reviewers firstly identified eligible articles by the abstracts available. Then, if the abstract met the inclusion criteria, the full article text was obtained **(** **Figure 1** **)**.

### Study Selection

Full articles were obtained if they met the following inclusion criteria: 1) published in English and on humans; 2) adult humans ≥18 years of age, free-living people, and all volunteers have been on the diets for more than 2 months; 3) control groups included; 4) one or more of the following lipids and lipoproteins assessed: HDL-c, TC, LDL-c, and TG. In addition, subject group characteristics, sex, age, BMI, sample size, location, recruitment procedure, and classifying information should be included. We found 12 studies that were consistent with our inclusion criteria (**Table 1**). Discarded citations included reviews, insufficient detail on classification, and other descriptive or intervention studies.

**Table 1:** Characteristics of literatures included in the meta-analysis and observational studies evaluating the effects of diets in HDL (mmol/l)^1^.

Study	Country	Cultural^2^	Design^3^	Median	BMI		Vegetarians		Omnivores		Weight
				age	Vegetarians	Omnivores	No. ofsubjects	Mean±SD	No. ofsubjects	Mean±SD	(%)
Thorogood *et al*. (1987)	UK	Euro-Amer	Cohort	38.2	–	–	1550	1.50±1.36	1198	1.49±1.20	12.0
Alexander *et al*. (1999)	USA	Euro-Amer	Cross	44.0	27.2±5.2	31.4±7.4	74	1.15±0.27	45	1.03±0.26	8.7
Krajcovicova *et al*. (2000)	Slovak	Euro-Amer	Cross	48.0	22.8±1.5	24.2±2.3	54	1.42±0.15	59	1.31±0.23	8.7
Li *et al*. (2001)	Australia	Euro-Amer	Cross	37.5	23.6±2.8	26.4±3.4	43	1.03±0.24	60	1.09±0.25	8.4
Hoffmann *et al*. (2001)	Germany	Euro-Amer	Cross	45.0	–	–	111	1.66±0.43	138	1.50±0.30	10.3
Robinson *et al*. (2002)	UK	Euro-Amer	Cross	30.0	24.7±5.2	24.7±5.2	43	1.21±0.33	43	1.47±0.47	7.9
Teixeira Rde *et al*. (2007)	Brazil	Asia-Latin	Cross	49.5	22.6±3.1	26.7±5.1	67	1.17±0.26	134	1.18±0.31	9.8
De Biase *et al*. (2007)	Brazil	Asia-Latin	Cross	35.8	23.5±4.5	25.4±5.2	19	1.43±0.38	22	1.45±0.47	5.8
Papadaki *et al*. (2008)	Greece	Euro-Amer	Cross	45.0	30.8±4.3	31.3±4.6	10	1.01±0.21	10	1.07±0.24	3.8
Chen *et al.* (2010)	China	Asia-Latin	Cross	52.0	22.9±2.9	23.3±3.5	173	1.44±0.34	190	1.61±0.38	10.8
K. Femandes *et al*. (2011)	Brazil	Asia-Latin	Cross	40.0	24.0±3.6	24.4±3.6	29	1.15±0.32	58	1.04±0.19	7.7
C. Juliana *et al*. (2013)	Brazil	Asia-Latin	Cross	37.0	23.0±1.6	23.5±1.2	18	1.09±0.20	29	1.16±0.31	6.1

1 Data were expressed as Mean ± SD; To convert from mg/dl to mmol/L, multiply by 0.02586;

2 Euro-Amer (Europe and North America): UK, USA, Slovak, Australia, Germany, and Greece; Asia-Latin (Asia and Latin America): Brazil and China.

3 Cross: cross-sectional studies; Cohort: cohort studies.

### Data Extraction

Two investigators independently extracted data and reached a consensus on all of the items. After the initial search procedures were completed, remaining citations were examined to determine whether they met the inclusion criteria. For articles that met the criteria, the following information was collected: country of origin of author, year of publication, country of origin of subjects, selection and characteristics of each diet group, demographics, and the mean HDL-c levels of a group of subjects while on that diet. Nutrient intake did not change within a study; any drift in the HDL-c level with time occurred simultaneously in both diet groups and therefore did not affect the differences in final levels.

### Data Synthesis and Analysis

**Figure 2:**
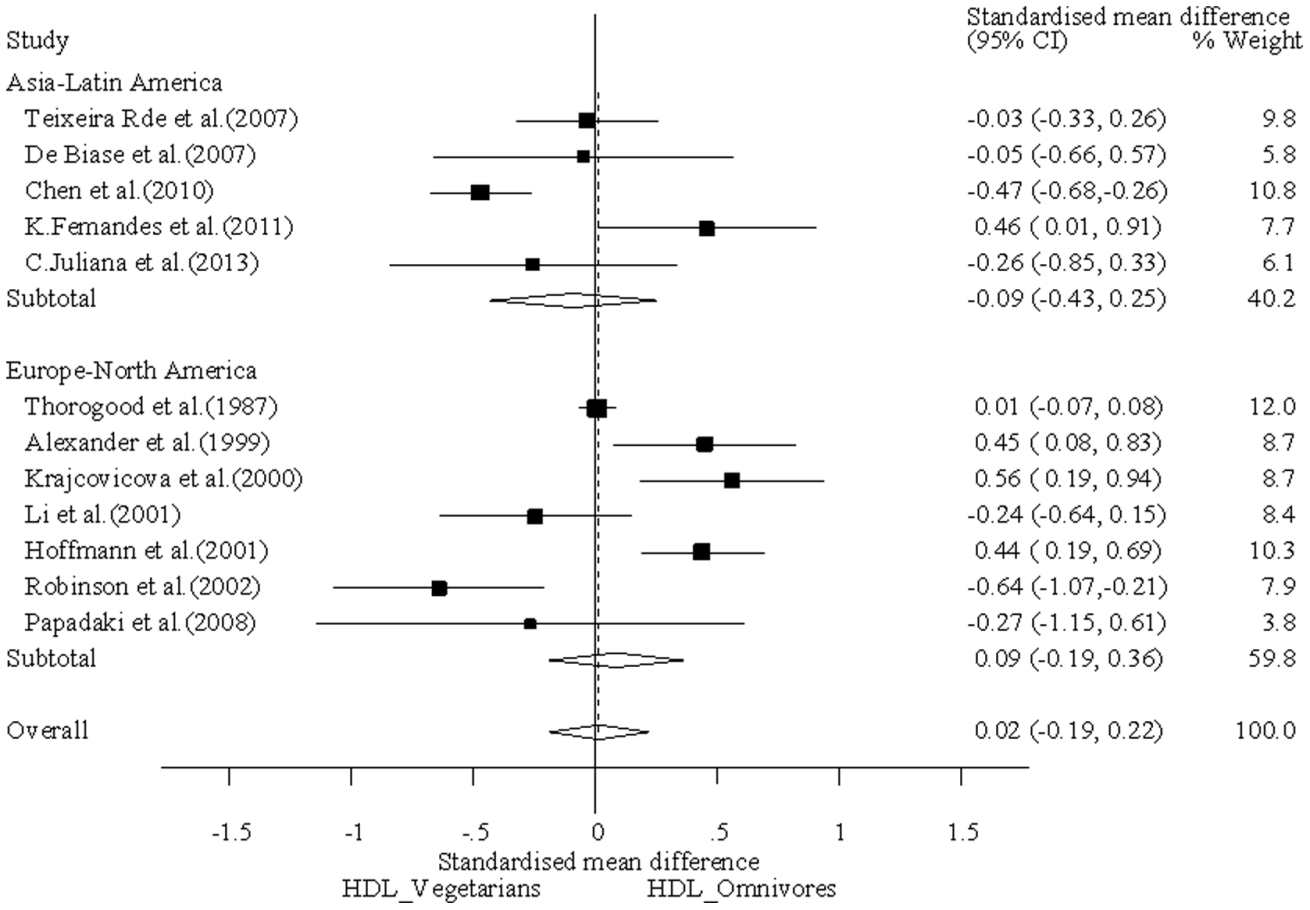
Forest plot of HDL-c levels among different diets. The squares and horizontal lines correspond to the study-specific SMD and 95% CI. The area of the squares reflects the study-specific weight (inverse of the variance). The diamond represents the pooled SMD and 95% CI. % weighted for random-effects.

**Figure 3:**
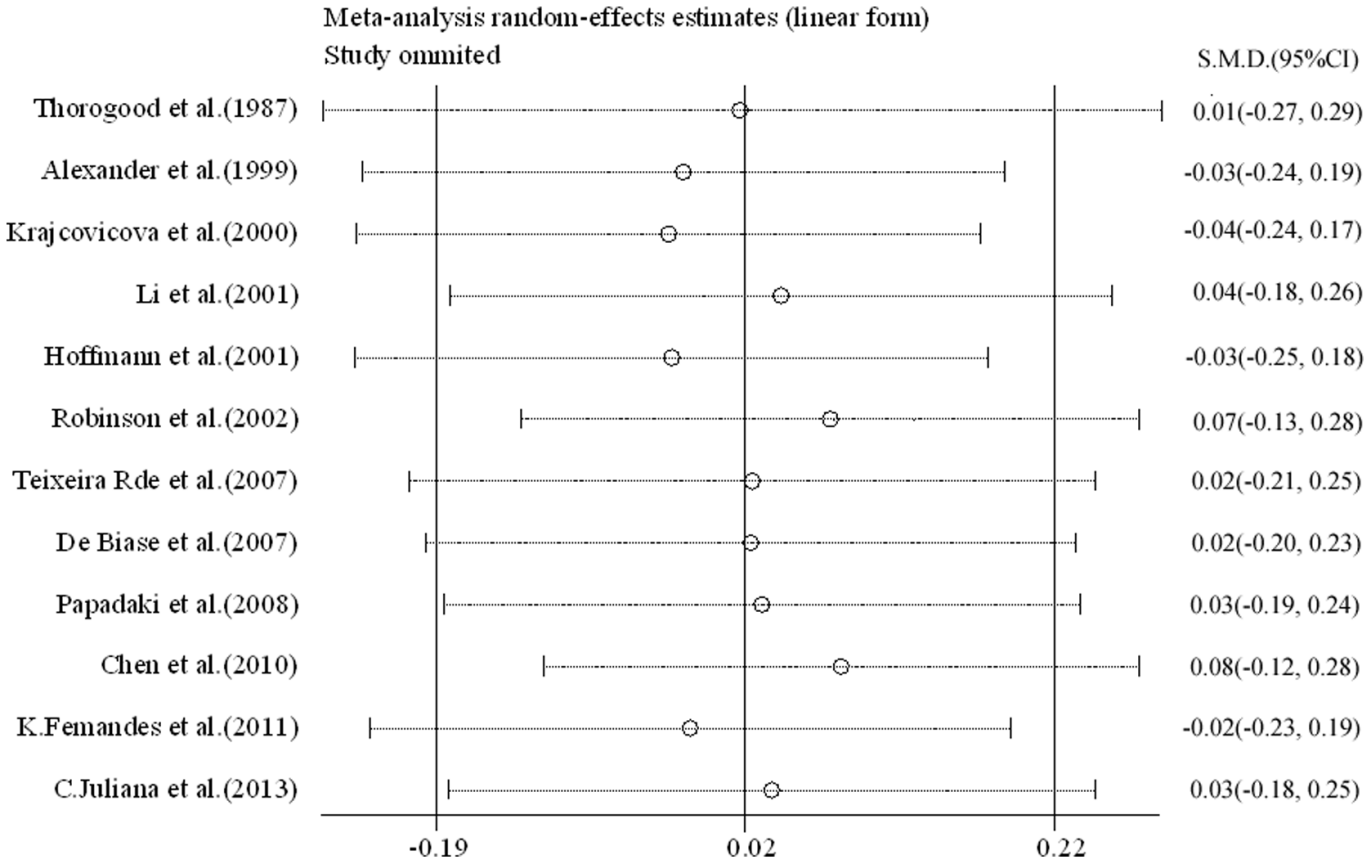
Sensitivity analyses of 12 studies, the part between two short lines shows the 95% CI of one study, each small circle indicates mean differences.

The strength of the association of HDL-c levels in different diets was measured as followed: Mean ± SDs (mmol/l) with 95% CI and % weight for all trials. To convert from mg/dl to mmol/l, multiply by 0.02586. Stratified analyses were also performed by some cultural similarities. A statistical test for heterogeneity was performed based on the Q-test [16] **(** **Figure 2** **)**, a *P* value greater than 0.05 indicates a lack of heterogeneity among studies. The summary SMD (Standardized Mean Difference) estimate was calculated by the random-effects model (the Der Simonian and Laird method was used) [17]. Funnel plots and Egger’s linear regression test were used to provide diagnosis of the potential publication bias [17] **(Figure S1; Figure S2)**. Sensitivity analyses were performed to assess the stability of the results **(** **Figure 3** **)**. All analyses were done with Stata software (version 10.0; StataCorp LP, College Station, TX), using two-sided *P* values.

## Results

### Results of the Literature Search

In the initial stage, 612 studies were included, of which 415 were excluded on the basis of screening using inclusion and discarded criteria. The remaining 197 abstracts were reviewed, and full reports were evaluated, of which 22 potentially relevant articles were included. Of the remaining studies, 10 additional studies were excluded, of these 12 trials published all-year time span that met specific inclusion criteria after a close examination **(** **Figure 1** **)**. Reports were excluded because age did not accord with, they did not report HDL-c levels, did not detail on classification, did not report human studies, and were not in English or English translations were unavailable. These studies included 4177 individuals, 2191 vegetarians and 1986 omnivores. The reviews were all observational studies (including cross-sectional studies and cohort studies).

### Study Characteristics

We identified 12 trials with 4177 individuals [5], [18]–[28]. Two types of diets were considered, and there were vegetarian diets and omnivorous diets. Vegetarians were classified as lacto-ovo vegetarians (people who consume eggs, milk, and dairy products), lacto-vegetarians (people who consume milk and dairy products, but not eggs), and ovo-vegetarians (people who consume eggs, but not milk or dairy products). Vegetarians consume no meat, or no more than six times per year, and they had to have been practicing their diets for more than 2 months prior to the study [29]. They have been recruited from the Vegetarian Society, Seventh-Day Adventist Churches, Adventist Seminary Institute, and monasteries. Omnivores were defined as people who eat food of both plant and animal origin [5]. They were from the surrounding communities, friends, relatives of vegetarians, and healthy public-service personnel or the Oxford arm of the European Prospective Investigation into Cancer and Nutrition (EPIC-Oxford) through advertisements, radio, posters, and others. In the present study, all volunteers ranged from 30 to 52 years old, without family history of overt vascular diseases (such as myocardial infarction, cerebrovascular, and angina etc), hyperlipoproteinemia, dyslipidemia, and related diseases.

### Effect of Vegetarian Diets and Omnivorous Diets on HDL-c Profile

In our study, the baseline characteristics of two groups were almost similar **(Table1)**. Results of this meta-analysis suggested that individuals consuming vegetarian diets, as a group, had the same HDL-c level as omnivores **(** **Figure 2** **)** (SMD = 0.02 mmol/l; 95% CI: −0.19 to 0.22 mmol/l). We assessed the association of plasma HDL-c levels between vegetarian and omnivores which stratified by some cultural similarities. In Asia and Latin America countries, it was suggested that there was no significant difference in plasma HDL-c level between vegetarians and omnivores (SMD = −0.09 mmol/l; 95% CI: −0.43 to 0.25 mmol/l), and in Europe and North America countries, the phenomenon was also consistent between the two diets (SMD = 0.09 mmol/l; 95% CI: −0.19 to 0.36 mmol/l). In light of this meta-analysis, we conclude that there is no evidence that plasma HDL-c levels differs in vegetarians and omnivores, even after adjusting for cultural circumstances.

Furthermore, we found no evidence of publication bias, and there was a good of heterogeneity among studies. However, there was a modest degree of sensitivity analyses because of two studies deviating a little far from SMD [23], [25], and they were kept for having no influence on heterogeneity **(Figure S1 and Figure S2)**. Differences in response between men and women were not all reported in the 12 studies, and therefore were not considered in the present report. But the present study’s results were almost applicable to both sexes, because there was no heterogeneity between them. Only 2 original studies [20], [30] excluded for gender classification, 2 references were studies of women [21], [31] and 1 of men [25], but there was an almost equal number of men or women in the two groups (in vegetarian group men and women were 673 and 1348, respectively; and in omnivores were 687 and 1016, respectively).

## Discussion

The present meta-analysis study provides evidence indicating that there was no significant difference in HDL-c levels between vegetarians and omnivores. Moreover, stratified analyses performed by cultural similarities also revealed that the plasma HDL-c levels were constant regardless of cultural circumstances. These results were against to our original expectation.

Initially, we hypothesized that omnivores consuming excess animal protein and saturated fatty acid had unbalanced diet and low HDL-c level, which was consistent with the notion of that omnivorous diets was associated with higher incidence of chronic cardiovascular diseases. However, based on the data produced in our meta-analysis study, the lack of meat in the diet seems not providing advantages to the vegetarians. In the included individual studies, because the only criteria to differentiate vegetarian and omnivore was meat consumption, the consumption of other animal products was ignored. As a mater of fact, majority of the vegetarians also consumes eggs, milk, and other dairies, and perhaps in larger amounts, thus reducing the contrast with omnivores. Egg yolk is one of the greatest cholesterol-containing foodstuffs; whereas milk and dairy products importantly contribute to the high saturated fat and also cholesterol content of the daily food intake. Omnivores usually take more proportion of lean meat instead of fat meat as their food; the lean meat itself does not increase plasma cholesterol levels [32]–[34]. Alcohol also increases HDL-c concentration and perhaps omnivores tend to consume more alcohol than vegetarians [35], [36]. In addition, vegetarians tend to take lower saturated fat and higher polyunsaturated fat than omnivores. Meta-analyses of metabolic ward studies [37] and controlled dietary trials [38] have revealed that saturated fat increases HDL-c, and so does polyunsaturated fat, although to a lesser extent. So, it is difficult to predict HDL-c level simply based on different diet groups. Consistently, previous reports [39], [40] also showed that diet and exercise interventions have no significant effect on HDL-c, but do affect measurements of other types of blood lipids.

Apart from environmental factors such as lifestyle and nutrition, about 70% of the variations in plasma HDL-c levels in humans are genetically determined [41], [42]. It is widely postulated that variations in plasma HDL-c concentration are determined by both the rate at which HDL-c is produced and the rate of catabolism of HDL particles [43], [44]. There are several known molecules that play a role in regulating plasma HDL-c concentration, including hepatic lipase (HL), lecithin cholesterol acyltransferase (LCAT), cholesteryl ester transfer protein (CETP), and phospholipid transfer protein (PLTP). It is now generally accepted serum HDL-c concentrations are regulated in part by members of the lipase enzyme family, especially HL, in which loss-of-function mutations are hypothesized to result in elevated HDL-c level. The lipases have highly conserved structural domains, and these enzymes function to metabolize triglycerides and phospholipids [43], [45]. It is important in the processing of lipids carried within lipoproteins and probably also in the uptake of lipoprotein particles into cells [46]. Although synthesized in nonendothelial cells, the secreted enzymes translocate to the surface of endothelial cells, where they carry out their metabolic function.

Lastly, we realize that there are several limitations in our study: 1. In our meta-analysis, we could not obtain information from all studies on the blood pressure, weight status, insulin or diabetes conditions which were main risk factors for dyslipidemia, and lacking of those original data limited our further evaluation of potential interactions. 2. Our results were based on unadjusted estimates, while more precise analysis should be conducted if more detailed individual data were available. 3. It was difficult to quantitatively describe the foods, especially all data were self-reported. 4. It was important to distinguish the differences between vegan diets and vegetarian diets. A vegan consumed no meat, eggs, and dairy products, but the latter included dairy products and eggs in the diets, which could produce different physiological effects.

In summary, our meta-analysis indicated that vegetarians and omnivores presented almost consistent HDL-c plasma levels regardless of different cultural background. A well designed population-based study with large sample sizes, detailed exposure information, and stratification for age, sex, location, race, and BMI is warranted to validate these findings.

## Supporting Information

Figure S1 Begg’s funnel plot for publication bias test. Each point represents a separate study for the indicated association SMD, standardized mean difference. Horizontal line means standard error.
(TIF)

Figure S2 Egger’s publication bias plot. Standardized effect estimates versus precision along with the regression line and the 95% CI about the intercept. Failure of this 95% CI to include zero indicates asymmetry in the funnel plot and may give evidence of publication bias. Guidelines include x = 0 and y = 0.
(TIF)

Checklist S1 PRISMA Checklist.
(DOC)
