# Supplementary figures and images for: Comparison of Vegetarian Diets and Omnivorous Diets on Plasma Level of HDL-c: A Meta-Analysis
Source: PLoS One. 2014 Mar 26;9(3):e92609. doi: 10.1371/journal.pone.0092609 (PMC3966789; doi:10.1371/journal.pone.0092609)

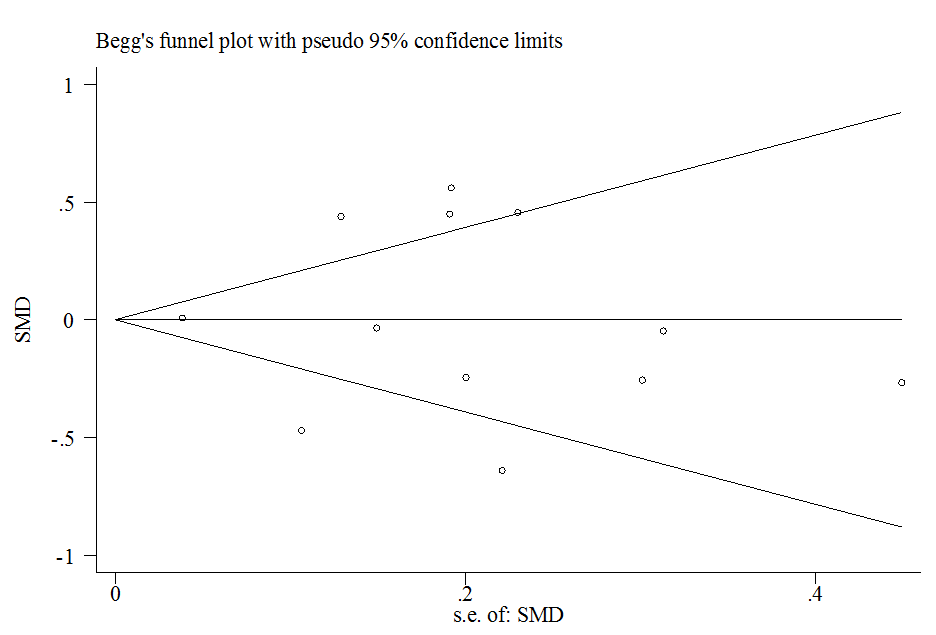

Supplement: Figure S1 — Begg’s funnel plot for publication bias test. Each point represents a separate study for the indicated association SMD, standardized mean difference. Horizontal line means standard error. (TIF) [file pone.0092609.s001.tif]

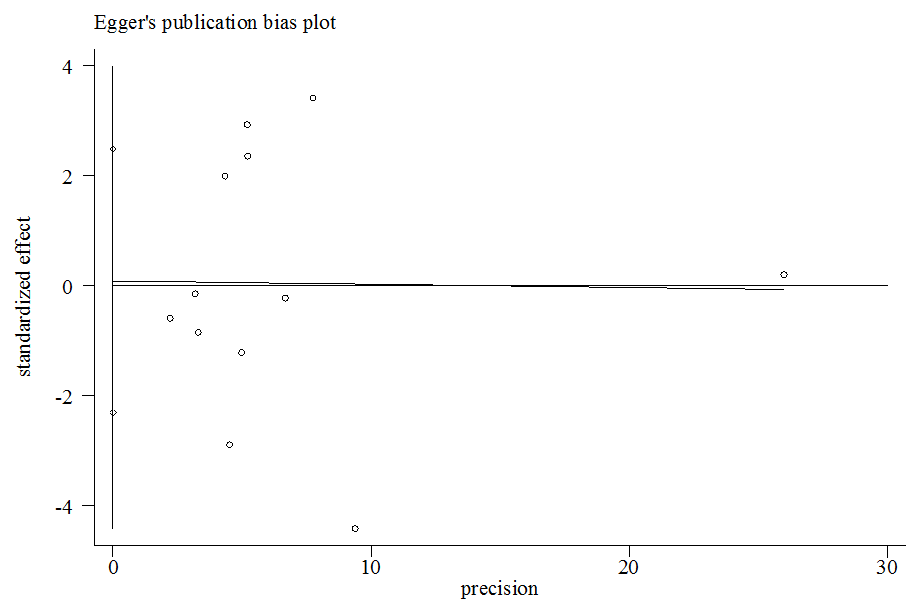

Supplement: Figure S2 — Egger’s publication bias plot. Standardized effect estimates versus precision along with the regression line and the 95% CI about the intercept. Failure of this 95% CI to include zero indicates asymmetry in the funnel plot and may give evidence of publication bias. Guidelines include x = 0 and y = 0. (TIF) [file pone.0092609.s002.tif]
